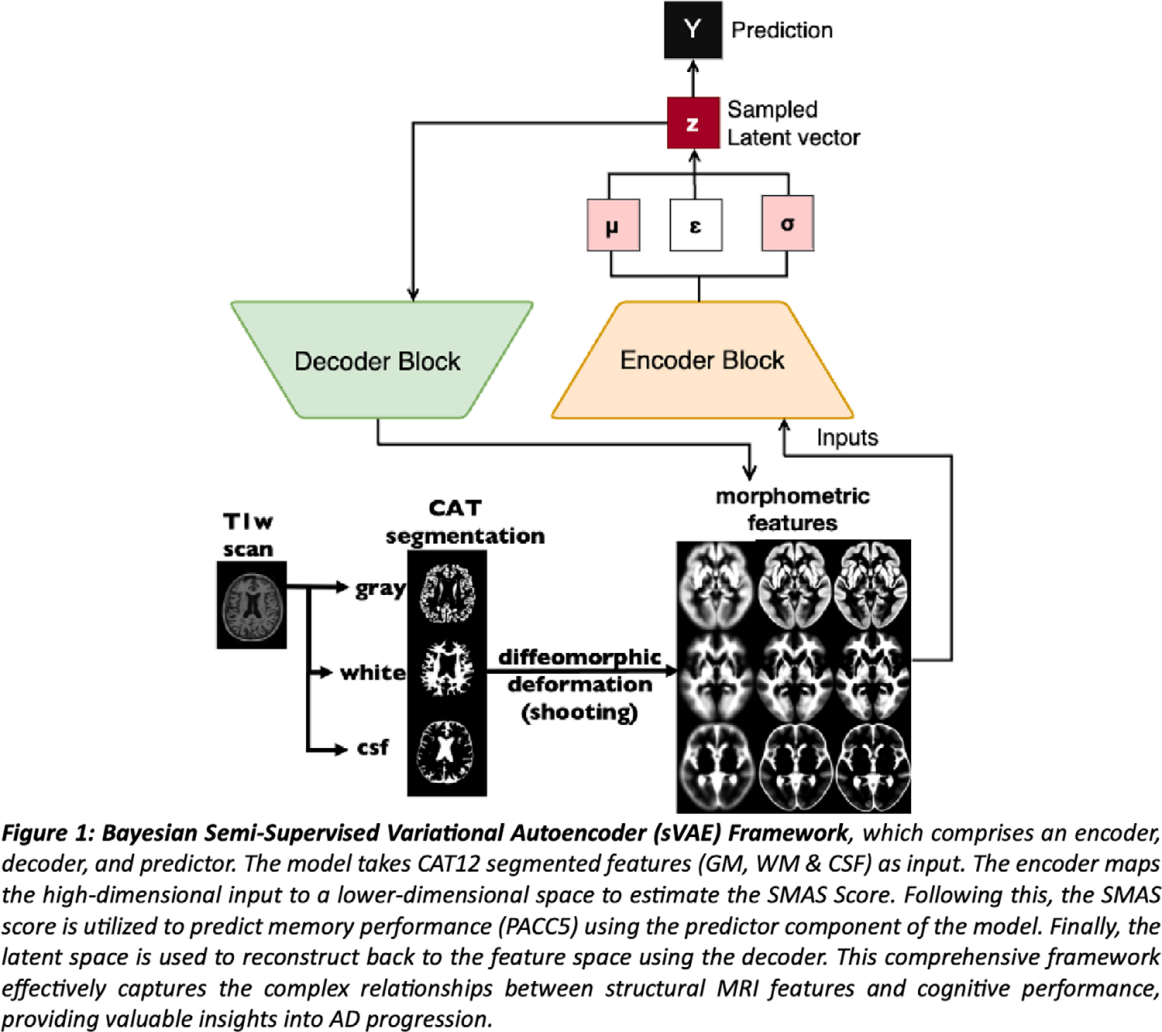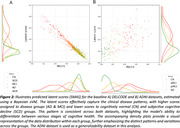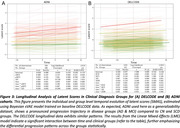# Structural MRI‐Based AD Score using Bayesian VAEs

**DOI:** 10.1002/alz.095270

**Published:** 2025-01-09

**Authors:** Aditya Nemali, Jose Bernal Moyano, Renat Yakupov, Hartmut Schütze, Annika Spottke, Alfredo Ramirez, Anja Schneider, Coraline D. Metzger, Christoph Laske, Daniel Bittner, Michael T. Heneka, Oliver Peters, Oliver Speck, Wenzel Glanz, Michael Wagner, Frank Jessen, Emrah Düzel, Gabriel Ziegler

**Affiliations:** ^1^ Institute of Cognitive Neurology and Dementia Research (IKND), Otto‐von‐Guericke University, Magdeburg Germany; ^2^ German Center for Neurodegenerative Diseases (DZNE), Magdeburg Germany; ^3^ Institute of Cognitive Neurology and Dementia Research (IKND), Otto‐von‐Guericke University Magdeburg, Magdeburg Germany; ^4^ German Center for Neurodegenerative Diseases (DZNE), Bonn Germany; ^5^ German Center for Neurodegenerative Diseases (DZNE), Bonn, Venusberg‐Campus 1, 53127 Bonn, Germany, Bonn Germany; ^6^ German Center for Neurodegenerative Diseases (DZNE), Tuebingen Germany; ^7^ Charité – Universitätsmedizin Berlin, corporate member of Freie Universität Berlin and Humboldt‐Universität zu Berlin, Berlin Germany; ^8^ German Center for Neurodegenerative Diseases (DZNE), Berlin Germany; ^9^ University of Bonn Medical Center, Dept. of Neurodegenerative Disease and Geriatric Psychiatry/Psychiatry, Venusberg‐Campus 1, 53127 Bonn, Germany, Bonn Germany

## Abstract

**Background:**

Alzheimer’s Disease (AD), a progressively worsening neurodegenerative disorder, impacts millions globally. Understanding its progression is crucial for developing effective interventions and management strategies. However, high variability in disease progression amongst individuals and the complexity of neuroimaging data pose significant challenges. Current diagnostic methods often fail to capture the nuanced progression, leading to delayed interventions, and lack uncertainty estimates, necessary for reliable decision‐making (Schaar et al., 2022). To address these issues, we here propose a Structural MRI‐Based AD Score (SMAS) using a Bayesian supervised Variational Autoencoder (Bayesian sVAE). The approach captures distinct morphometrical brain patterns associated to cognitive impairment across stages of AD. The score and model has potential to support personalized treatment plans and improve the efficacy of diagnostic tools.

**Method:**

We used 415 and 200 longitudinal MRI scans from DELCODE (Jessen et al., 2018;) and ADNI (Salvatore et al., 2018), respectively. We focused on features computed from T1‐w images using CAT12 longitudinal pipeline (Gaser et al., 2022). The Bayesian sVAE model (Fig. 1) consists of an encoder network that learns to map brain features into a lower‐dimensional embedding space. Moreover, a predictive block predicts memory performance based on the latent mapping, and a decoder block that reconstructs the original input images from the latent space. We trained the sVAE on baseline DELCODE cohort with the expectation that the model will capture brain signatures characterizing aging and progression towards AD. We then assessed its generalization ability on follow‐up data and on ADNI.

**Result:**

The Bayesian sVAE model, trained on sMRI features, shows potential for a parsimonous anatomy‐based characterization of individual disease progression. The model’s capacity to differentiate among various clinical groups using a single score (SMAS), and its correlations with key clinical and demographic variables, suggest its potential for identifying structural brain related to progression (Fig. 2). Using longitudinal test data, the model‐based latent scores indicated expected group differences and changes (Fig. 3).

**Conclusion:**

The Bayesian sVAE model has shown promise in continuously monitoring atrophy over stages of AD progression. We provide indications for generalizability to unseen data (ADNI), suggesting robustness across different imaging protocols and scanners, and considerable stability over repeated measures.